# Optimization of Nanosubstrates toward Molecularly Surface-Functionalized Raman Spectroscopy

**DOI:** 10.1021/acs.jpcc.2c03524

**Published:** 2022-08-08

**Authors:** Paulo De Carvalho Gomes, Mike Hardy, Yazmin Tagger, Jonathan James Stanley Rickard, Paula Mendes, Pola Goldberg Oppenheimer

**Affiliations:** † School of Chemical Engineering, College of Engineering and Physical Sciences, University of Birmingham, Birmingham B15 2TT, U.K.; ‡ Department of Physics, Cavendish Laboratory, University of Cambridge, JJ Thomson Avenue, Cambridge CB3 0HE, U.K.; § Healthcare Technologies Institute, Translational Medicine, Mindelsohn Way, Birmingham B15 2TH, U.K.

## Abstract

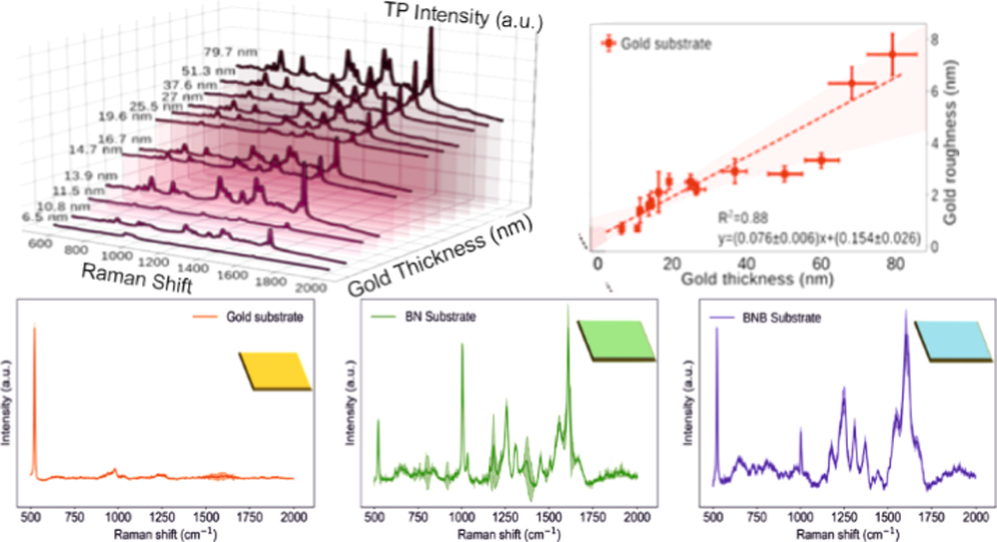

Diagnostic advancements require continuous developments of reliable analytical sensors, which can simultaneously fulfill many criteria, including high sensitivity and specificity for a broad range of target analytes. Incorporating the highly sensitive attributes of surface-enhanced Raman spectroscopy (SERS) combined with highly specific analyte recognition capabilities via molecular surface functionalization could address major challenges in molecular diagnostics and analytical spectroscopy fields. Herein, we have established a controllable molecular surface functionalization process for a series of textured gold surfaces. To create the molecularly surface-functionalized SERS platforms, self-assembled benzyl-terminated and benzoboroxole-terminated monolayers were used to compare which thicknesses and root-mean-square (RMS) roughness of planar gold produced the most sensitive and specific surfaces. Optimal functionalization was identified at 80 ± 8 nm thickness and 7.2 ± 1.0 nm RMS. These exhibited a considerably higher SERS signal (70-fold) and improved sensitivity for polysaccharides when analyzed using principal component analysis (PCA) and self-organizing maps (SOM). These findings lay the procedure for establishing the optimal substrate specifications as an essential prerequisite for future studies aiming at developing the feasibility of molecular imprinting for SERS diagnostic applications and the subsequent delivery of advanced, highly selective, and sensitive sensing devices and analytical platforms.

## Introduction

Sensing via molecular functionalization can provide a superior analytical selectivity that has the potential to advance the biomedical, chemical, and biological sciences for a breadth of applications ranging from catalysis,^1−8^ through sensors,^9−14^ drug delivery,^15−20^ and to chromatographic separation.^21−23^ An example of such molecular functionalization is surface molecular imprinting, which involves in situ surface polymerization of functional monomers in the presence of a template molecule extracted afterward. The polymerization leaves synthetic receptors within nanocavities on surfaces complementary in size, shape, and functional group orientation to the target template.^13,24^ Target affinity and selectivity in functionalized recognition platforms are achieved via multiple surface interactions that combine hydrogen bonding, van der Waals forces, electrostatic interactions, or covalent bonding.^25−32^

For detection applications, surface functionalization alone does not meet the requirements for a sensor without some form of a transduction technique applied to convert the molecular interaction into a measurable signal. Surface-enhanced Raman spectroscopy (SERS) can be used for this purpose. It is a highly sensitive biochemical sensing technique, enhancing the Raman signal of molecules adsorbed on a nano-roughened noble metal surface, where the incident light excites surface plasmon-polaritons. SERS yields characteristic molecular fingerprints of vibrational spectra, enabling a rapid, high-sensitivity sensing transduction method. Thus, it is crucial to establish the optimal gold thickness and nano-roughness that allow the most selective molecular surface functionalization and the highest possible SERS effect. While gold or silver nanoparticles, colloids, and nanopores have been explored for potential surface functionalizations,^32−38^ planar gold substrates, which can be more stable and reproducible molecularly functionalized substrates coupled with SERS, have not been studied. More recently, researchers have examined the effect of the pH, solvents, and polymer concentration on planar gold surface functionalization.^39−42^ However, there has been a lack of information on the effects of surface roughness associated with planar gold thickness impacting both the functionalization and the achievable SERS signals.

This work systematically evaluates the influence of a broad range of gold thickness layers and roughness parameters on the SERS effect and the molecular surface functionalization. The biochemical interpretation of the Raman spectral data constitutes a solid basis for establishing the optimal substrates as an essential prerequisite for future studies to develop the feasibility of molecular imprinting for SERS applications. Experimentally, to study and optimize the SERS detection on functionalized gold surfaces, two types of self-assembled monolayers (SAM) were employed (Figure S1). Both functionalizations have an acrylamide-terminated SAM, typically used as a foundation to architect the molecularly imprinted surfaces. The variations between the SAM are in the functional group. One has a benzoboroxole (BNB) functional group, which binds to carbohydrates via reversible covalent bond formation with the carbohydrate hydroxyl groups.^43^ A second has a benzyl functional group that does not bind to carbohydrates.^13,14^ The difference in selectivity binding of the BNB-terminated SAM and the benzyl-terminated SAM, combined with different planar gold thickness and roughness, is demonstrated using a trisaccharide (melezitose (Mel)). An optimal functionalization was established for 80 nm thickness and 3.5 nm root-mean-square (RMS) gold substrates with enhanced SERS signal and analyte sensitivity.

Future sensor design would significantly benefit from fabricating the optimized gold substrates and will further enable the controllable fabrication of the desired platforms for multiplex biosensing. The unique combination of surface functionalization and SERS will also lay the platform for designing advanced substrates with tunable localized plasmonic fields for various chemical, biological, and lab-on-chip sensing applications.

## Materials and Methods

### Materials

Silicon wafers were purchased from Si-Mat. Ethanol and methanol were purchased from Sigma-Aldrich and used without further purification. Melezitose was purchased from Acros Organics. The benzyl-terminated SAM molecule—3,3′-disulfanediylbis(*N*-phenylpropanamide) and benzoboroxole-terminated SAM molecule—3,3′-disulfanediylbis(*N*-(1-hydroxy-1,3-dihydrobenzo[*c*] oxaborol-6-yl)propanamide)^13^ were synthesized in-house, as previously reported.^13^ The acrylamide-terminated SAM molecule—*N*,*N*′-bis(acryloyl)cystamine was purchased from Sigma-Aldrich. A gold target (57 mm in diameter and a thickness of 0.1 mm) for sputtering was ordered from Agar Scientific.

### Substrate Preparation

A standard 6 in. silicon wafer ⟨100⟩ was cut into square pieces of 1 × 1 cm^2^ using a diamond scribe. These silicon substrates were then placed on a heating plate at 250 °C for 1 min and subsequently cleaned several times using a CO_2_ snow jet gun.^44^ Following the cleaning step, the samples were placed in the sputter coater. An Agar automatic sputter coater was used to create gold films over silicon. A range of gold thickness and roughness was accomplished by varying the sputter times and the currents used. The inner chamber argon gas pressure was kept at 0.08 mbar throughout all of the depositions, with the reaction time varying from 20, 40 to 80 s and current of 10, 20, 30, and 40 mA. The gold film surface was further tuned by adjusting the radial distance of the samples in the sputter coater platform. Changing the radial positions allowed to consistently achieve a span of multiple grain sizes and roughness for the same thicknesses.

### Atomic Force Microscopy (AFM)

A JPK NanoWizard II atomic force microscope (AFM) was used to characterize the surfaces’ height, roughness, and grain size. The AFM measurements were performed using tapping mode via an intermittent contact mode of the cantilever tip, with the sample in ambient conditions. NCHV-A cantilevers with a resonance frequency of 320 kHz and stiffness of 42 N m^–1^ were used.^45^ Height, roughness, grain size, and phase images were analyzed with Gwyddion software (version 2.55).

### Molecular Surface Functionalization

Gold-coated silicon substrates were functionalized individually with different molecules, benzyl-terminated SAM, and benzoboroxole-terminated SAM. Initially, a 1 mM solution of benzyl-terminated SAM molecules in methanol was prepared and followed by a separate 1 mM solution of benzoboroxole-terminated SAM molecules in ethanol.^13^ The gold-coated samples were subsequently immersed in the different solutions for 24 h and dried under argon gas (Ar). Further gold-coated silicon substrates were functionalized with 0.1 mM thiophenol (TP) solution diluted in ethanol for 2 h. Nonfunctionalized bare gold-coated silicon substrates were used as references. Following each functionalization procedure, the samples were immediately analyzed via Raman spectroscopy.

### Raman Spectroscopy

Raman spectra were collected using a Renishaw inVia Qontor confocal Raman microscope equipped with a 633 nm laser. The spectra were typically acquired at a 10 s exposure time and one accumulation or 3 s exposure time with three accumulations and a laser power of 10 mW with a 633 nm laser to avoid photochemical effects in the spectra, sample damage, or degradation. A 50× objective with a 0.75 numerical aperture was used for measurements over a range of 500–2500 cm^–1^ relative to the excitation wavelength.^45^ An intelligent-fitting filter was applied for baseline subtraction. Data analysis from the acquired spectra was performed using Python-written algorithms. The peak intensities for prominent peaks throughout this work were gathered and later analyzed from the raw data following the baseline subtraction. The melezitose affinity tests were performed using a 785 nm laser.

### Code Availability

The customized written Python algorithm can be downloaded from https://github.com/PauloAxcel/MIP-data-analysis/blob/master/plot_test.py.

### Ellipsometry

Ellipsometry data was collected using J.A. Woollam α-SE Ellipsometer with a wavelength range of 380–900 nm and three angles, 65, 70, and 75°, with a 10 s acquisition between the angles. Three measurements were acquired per sample while ensuring that the position of the light spot changed for each measurement. The data from the ellipsometer was analyzed using the CompleteEASE Software. The analysis for a standard substrate was performed by defining the substrate with a B-spline and 0.1 eV resolution. The B-spline fitted the PHI and DELTA functions of all three angles together, acquired during the measurements of the substrate. The fit yielded both the refractive index, *n*, extinction coefficient, *k*, and roughness per measurement.^13^ Subsequently, the analysis proceeded to the functionalized substrate by adding a new layer to the B-spline model. The layer added was a Cauchy function capable of fitting the functionalized layer thickness to the new PHI and DELTA values.

### Carbohydrate Preparation

A range of melezitose concentrations (0.001–10 mM) was prepared and diluted in deionized (DI) water. A first dilution test was performed using an aluminum substrate, where a drop of 0.001–10 mL was dry-cast over the surface and, once dried, analyzed using Raman spectroscopy. Subsequently, using a 10 mM melezitose solution, the different molecular surface functionalization substrates were immersed for 30 min, dried under argon, and immediately analyzed using Raman spectroscopy. A comparison between the substrate before and after immersion in the carbohydrate solution was established using principal component analysis (PCA) and self-organizing maps (SOMs). The classification tests were performed using the *k*-nearest neighbors (*k*-NN).

### Multivariate Analysis

Multivariate analysis^46^ was performed using the self-optimizing Kohonen index network (SKiNET) based on self-organizing maps (SOMs), described in detail in ref (47). SOMs are single-layer artificial neural networks represented as a two-dimensional (2D) hexagonal array of neurons. Inspired by the visual cortex in the brain, the SOM is trained so that neighboring neurons activate according to similar inputs, in this case, Raman spectra. Each neuron has a weight vector with a length equal to the number of variables in a spectrum. Through exposing the network to training samples over several iterations, the weights are gradually adjusted to be similar to the input data so that each neuron only activates on a given spectral signature. The result is a projection of hyperspectral data into 2D space that can be shown as visible clustering according to type, group, and state. SKiNET employs the self-organizing map discriminant index (SOMDI), which appends a set of label vectors to each neuron and allows us to study the most prominent features that cause the activation of a particular neuron to a class label. Subsequently, a supervised learning step is introduced to optimize the network, and the class label associated with each neuron is used to quickly identify new data presented to the SOM.

### Principal Component Analysis (PCA)

The minute differences in spectra between the two stages were interpreted using another multivariable analysis method. The analysis included the cluster separations of the different stages, i.e., Raman spectra of the sample prior to and post sugar submersion. PCA analyses were used with the Euclidean distance to infer how the separated clusters separated at each stage. The Euclidean distance between different cluster centroids was initially calculated by fitting an ellipsoid to the cluster. Subsequently, the ellipsoid center position of principal components (PC1) and (PC2) was identified and used to calculate the Euclidean distance between prior to and post the carbohydrate submersion clusters (eq 1)
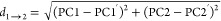
1

PC and PC′ correspond to the different cluster centers, and the distance error is considered proportional to the ellipse’s major and minor axis. The cluster distance was compared for the varying molecular surface functionalization and thicknesses. For the PCA analysis, PCA loadings are calculated based on the eigenvectors and eigenvalues obtained from the matrix operations to find the principal components (PC). The loadings have specific information related to the initial Raman data, and depending on the PC space, the loadings can have different features. In a 2D PCA system, the different PC quadrants’ loadings are expressed as PC1 > 0 and PC2 > 0, PC1 < 0 and PC2 > 0, PC1 < 0 and PC2 < 0, and PC1 > 0 and PC2 < 0 obtaining four different loading fingerprints related to the four quadrants.

### COMSOL

COMSOL Multiphysics 5.3 was used to simulate the enhancing factors of the substrates. The analysis was achieved by processing the 2D AFM images acquired and putting them into COMSOL by formatting the surface morphology image into a readable format. The AFM height profile data points were later plotted and saved in an SVG file format. Later, the images were converted into a DXF file format, which COMSOL can read. Four different profile slices were taken per AFM measurement per thickness and run on the COMSOL wave optics package. The scattering field was simulated after the incidence of a plane wave over the surface. The plane wave was modulated with the Raman wavelength used for the optical measurements (633 nm). The enhancing factor (*E*/*E*_0_)^4^ was calculated over the encapsulating region above the surface profile, and the maximum value was recorded and later processed.

## Results and Discussion

Molecular surface functionalization was accomplished via the kinetic and thermodynamic self-assembly pathways on top of a range of gold thicknesses, i.e., 5–80 nm (Figure S1A-i), where the benzyl-terminated SAM (BN) and benzoboroxole-terminated SAM (BNB) (Figure S1A-ii,iii) were formed. Figure S1B shows the characteristic Raman fingerprints for each molecule in the molecular surface functionalization steps, including the plain gold surface (Figure S1B-i), the benzyl-terminated SAM (Figure S1B-ii), and benzoboroxole-terminated SAM (Figure S1B-iii)-functionalized gold surfaces. The characteristic peaks for each functionalization are summarized in Figure S2.

Establishing the optimal gold thickness for improved functionalization coupled with the SERS effect is vital for further implementing molecularly imprinted surfaces for advanced spectroscopic sensing platforms. We have thus fabricated a range of substrates with varying gold thickness (5–80 nm) and roughness (Table 1), which were characterized using atomic force microscopy (AFM), ellipsometry (Figure S3), and Raman spectroscopy (Figure 2A). Initially, no baseline subtraction was applied to the spectra, assessing how the background signal varies with gold thickness. The area under the curve (AUC) was found to increase with the increasing gold thickness (Figure S4A) attributed to the increased fluorescence, while the main silicon peak (520.5 cm^–1^) decreased exponentially with an increasing gold thickness (Figure S4B) due to the attenuation of the substrate Raman signature with the thicker gold layers. Thiophenol (TP) was used as the probe molecule for the spectroscopic analysis due to its strong SERS fingerprint, enabling an overall comparison between gold thicknesses.

**Table 1:** AFM Measurements over the Gold Thickness and Mean Square Roughness (*R*_RMS_ or *R*_q_), Followed by SERS Measurements of Different Functionalizations^a^

Au thickness (nm)	Au *R*_rms_ (nm)	TP intensity/counts (mW^–1^)	BN intensity/counts (mW^–1^)	BNB intensity/counts (mW^–1^)
6.5 ± 0.6	0.7 ± 0.2	245 ± 15	39 ± 6	33 ± 5
10.8 ± 0.9	0.7 ± 0.1	4205 ± 651	249 ± 15	124 ± 11
11.5 ± 0.9	1.4 ± 0.5	1277 ± 571	122 ± 11	141 ± 11
13.9 ± 0.6	1.6 ± 0.4	12 358 ± 778	151 ± 12	175 ± 13
14.7 ± 1.0	1.7 ± 0.2	5602 ± 443	131 ± 11	176 ± 13
16.7 ± 2.4	2.1 ± 0.8	8669 ± 500	207 ± 14	210 ± 14
19.6 ± 1.0	2.5 ± 0.3	2707 ± 247	126 ± 11	141 ± 11
25.5 ± 1.4	2.5 ± 0.2	5607 ± 305	220 ± 14	229 ± 15
27.0 ± 2.5	2.2 ± 0.2	9049 ± 469	166 ± 12	84 ± 9
37.6 ± 3.5	2.9 ± 0.5	6920 ± 345	252 ± 65	205 ± 53
51.3 ± 6.7	2.8 ± 0.4	11 263 ± 584	235 ± 28	269 ± 26
79.7 ± 8.0	7.2 ± 1.0	17 237 ± 910	1748 ± 84	1244 ± 58

a Gold thickness and roughness were obtained from three different samples and ten measurements per sample. The last three columns describe the maximum measured SERS signal of the substrates functionalized with TP, benzyl-terminated SAM (BN), and benzoboroxole-terminated SAM (BNB). The intensity was average, and the standard deviation was calculated (*n* = 100), followed by a spectral band measure, for TP, at 1618 cm^–1^ and for BN and BNB 1000 cm^–1^.

**Figure 1:**
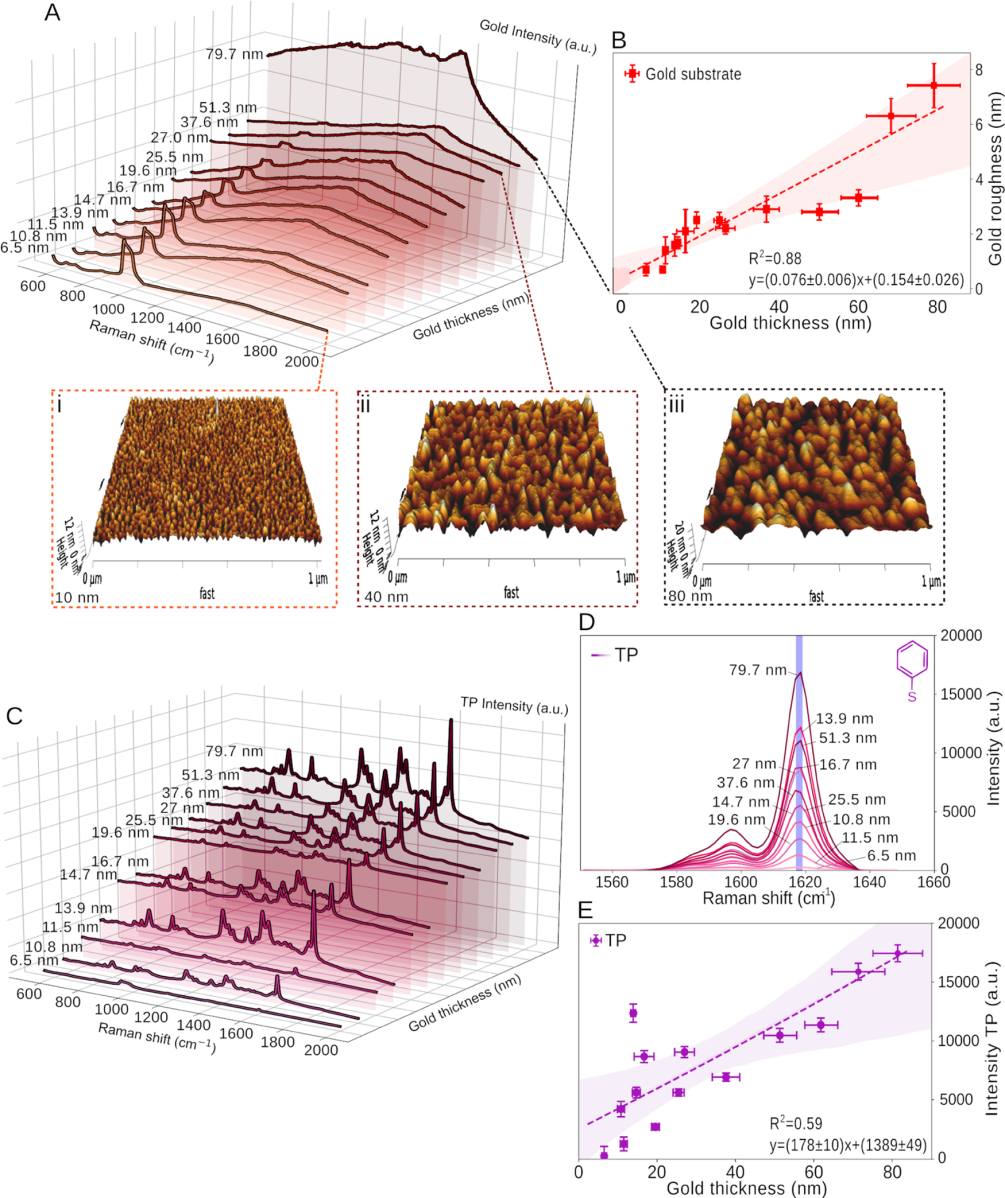
(A) Waterfall SERS spectra of planar gold surfaces as a function of the increasing thickness. Three-dimensional (3D) AFM height images of gold-coated silicon substrates with a thickness of (I) 10 nm, (ii) 40 nm, and (iii) 80 nm. (B) Roughness vs gold thickness acquired from the AFM measurements. (C) Waterfall Raman spectra of 0.1 mM thiophenol (TP) over gold substrates with a range of thicknesses. (D) Representative SERS spectra of the 1600 cm^–1^ peak of thiophenol (TP) as (E) a function of the increasing gold thickness, resulting in a positive linear relationship between the thickness and the acquired SERS signal.

**Figure 2:**
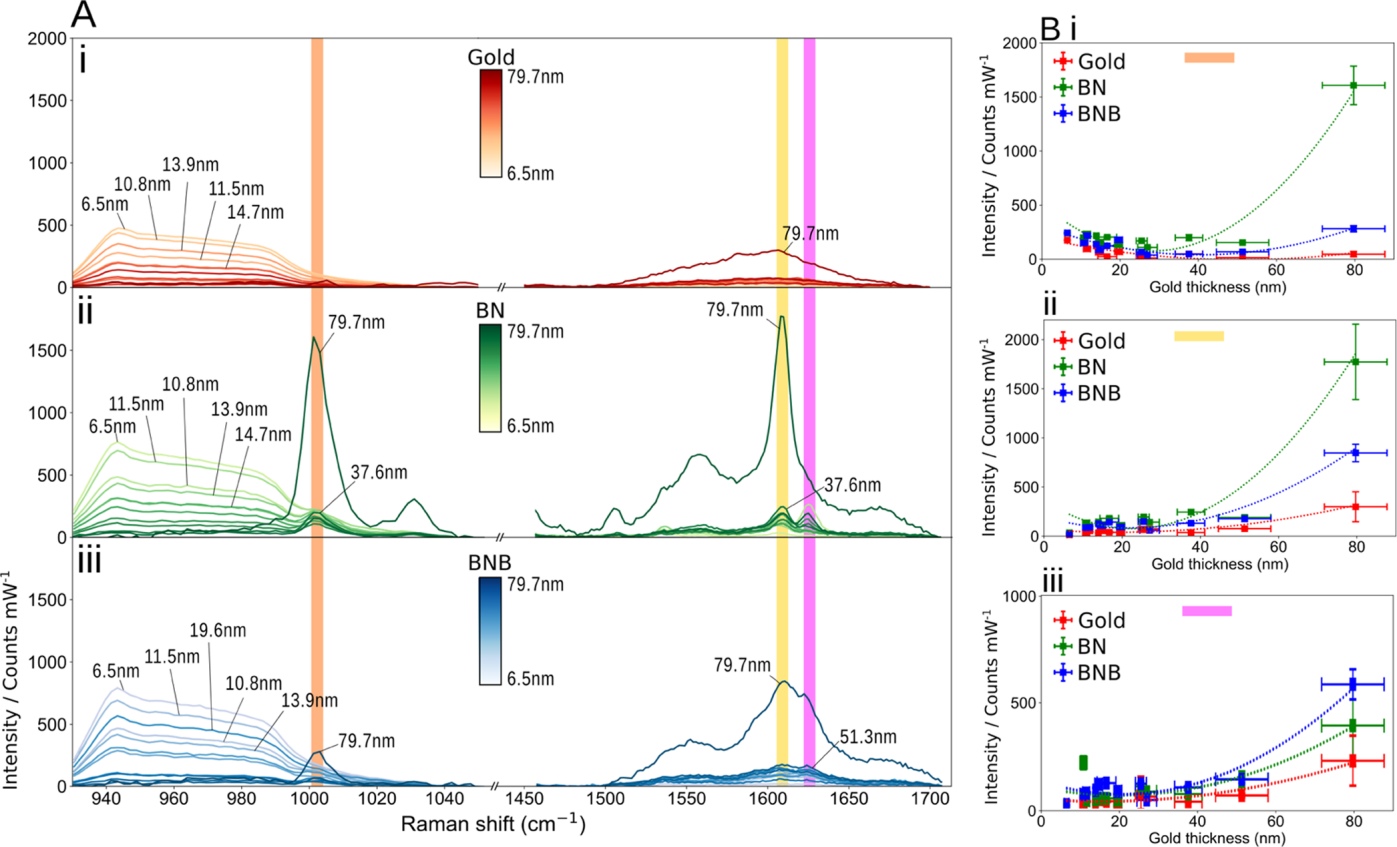
(A) Raman spectra for gold, benzyl-terminated SAM (BN), and benzoboroxole-terminated SAM (BNB) as a function of the gold thickness. Selected Raman regions for (i) planar gold, (ii) BN, and (iii) BNB at 1000 cm^–1^ (orange), 1605 cm^–1^ (yellow), and 1630 cm^–1^ (pink), highlighting the most significant peaks relative to the gold thickness. (B) Variation of the signal intensity for planar gold, BN, and BNB at peaks of ((i) orange) 1000 cm^–1^, ((ii) yellow) 1605 cm^–1^, and ((iii) pink) 1630 cm^–1^.

Three reproducible samples were fabricated for each gold thickness, and each was measured ten times via the AFM to acquire an average gold thickness and RMS roughness. The results revealed that as the gold thickness increased, the *R*_RMS_ also increased (Figures 1B and S5A–C). Additionally, the average film grain size for the different gold thicknesses measured in Table 1 also increased, obtaining values of 11.7 ± 3.4, 14.7 ± 3.8, 14.3 ± 3.7, 16.2 ± 4.1, 17.9 ± 4.2, and 21.3 ± 4.6 nm for 10, 20, 30, 40, 60, and 80 nm gold thicknesses, respectively. For Raman spectral analysis, each sample was measured via mapping of 100 points, with the maximum intensity per functionalization being tracked, averaged, and the standard deviation calculated (Table 1). Peak intensities at 1000 cm^–1^ for benzyl-terminated SAM and benzoboroxole-terminated SAM (Figure 2A-ii,iii) and 1618 cm^–1^ for thiophenol (Figure 1D) were recorded. The highest intensity was measured for 80 nm films with an overall 70-fold increase in intensity between 5 and 80 nm gold thickness (Figure 1E). Roughened platforms are well-known to make good SERS substrates, and a linear relationship between gold thickness and roughness for the sputter-coated gold substrates was established from the AFM measurements (Figure 1B), where an 8-fold increase in *R*_RMS_ is observed, 5–80 nm. Given the positive correlation between surface roughness and gold grain sizes,^48^ a relation between grain size from the roughness and thickness could be further extrapolated (Figure S5). We have examined the optical properties (*n*, *k*) of the pristine and functionalized gold films by ellipsometry (Figure S3A–E). The calculated gold roughness from the ellipsometry measurements is 8.9 ± 3.1 nm, which is in good agreement with the value obtained from the AFM measurements (Figure 1B) for the 80 nm gold layer. Subsequently, an in-depth systematic analysis of the influence of gold thickness on the achievable SERS signal with the functionalized benzyl-terminated SAM and benzoboroxole-terminated SAM was carried out and compared to the sputtered gold (Figure 2). From the ellipsometry analysis, we have found the real part of the index of refraction, *n*, and the extinction coefficient, *k*, for gold, benzyl-terminated SAM, and benzoboroxole-terminated SAM surfaces (Figure S3A–E).

The signal intensity variation of gold, benzyl-terminated SAM, and BNB-terminated SAM as a function of the increasing gold thickness was subsequently studied for representative peaks of interest at 1000 cm^–1^ (Figure 2A, orange), 1605 cm^–1^ (Figure 2A, yellow), and 1630 cm^–1^ (Figure 2A, pink). The corresponding peak assignments to these are summarized in Table 2.

**Table 2:** Inflection Points (IP) for the Characteristic Peaks for the Different Molecular Surface Functionalization, Gold, Benzyl-Terminated SAM (BN), and Benzoboroxole-Terminated SAM (BNB), and Their Tentative Assignments

	IP			
Raman peak cm^–1^	gold	BN (nm)	BNB (nm)	peak assignment
1000	60 ± 2 nm	40 ± 5	50 ± 5	*v*(C–C) aromatic ring vibrations^73^
1605	none	35 ± 3	35 ± 3	C–C stretching of the aromatic ring^72^
1630	none	30 ± 2	30 ± 2	*v*(C=O) vibration^72^

Interestingly, we have observed an intensity inflection point (IP) associated with the signal variation as a function of the gold thickness, where the signal trend changes from decreasing to increasing (i.e., the derivative of the signal trendline changes sign) as a function of the increasing gold thickness (Figure 2), predominantly appearing on the functionalized gold surfaces. The average IP is found at 45 ± 5 nm for 1000 cm^–1^, 35 ± 1 nm for 1605 cm^–1^, and 28 ± 3 nm for 1630 cm^–1^ peaks (Figure S6). For the peak at 1000 cm^–1^, associated with the aromatic ring vibration, the two SAM exhibit IPs with the signal initially decreasing until reaching a thickness of 40 ± 5 nm for benzyl-terminated SAM and 50 ± 5 nm for BNB-terminated SAM and inverting this trend past these minima (Figure 2B-i). Similar behavior is observed for the peak at 1605 and 1630 cm^–1^, where both SAM signals start to increase when the 35 ± 3 nm gold thickness value is reached (Figure 2B-ii,iii). The Raman bands present in the two SAM spectra (Figure 2A-ii,iii) and the 1600 cm^–1^ band in the thiophenol spectrum (Figure 1C) are attributed to the corresponding molecules. As a phonon mode associated with the interaction of light with the underlying silicon layer, we also observed a monotonic decrease in signal intensity of the silicon at 520 and 940 cm^–1^ with the increasing gold film thickness (Figures S4B and 2A). The impinging photons must traverse greater distances to interact with the silicon at the substrate interface, thus attenuating the silicon Raman signal.^49^

The Raman signal trends for standard thiophenol analyte and the benzyl-terminated SAM and BNB-terminated SAM functionalization molecules can be compared via the study of the similarly placed de-excitation bands for the respective molecules. Thus, we monitor Raman scattering events that have similar wavelengths such as 1618 cm^–1^ for thiophenol and 1605 cm^–1^ for both benzyl-terminated SAM and BNB-terminated SAM bands. This similarity in de-excitation wavelength highlights molecular effects arising from preferential surface adsorption or steric effects^50^ rather than resonant plasmonic enhancement effects.^51,52^ While thiophenol and molecular functionalization molecule plots appear qualitatively similar, we fit the 1618 cm^–1^ thiophenol band with a linear line (Figure 1E) (albeit with outliers in the sub-20 nm range) and adjudge benzyl-terminated SAM and benzoboroxole-terminated SAM at 1605 cm^–1^ best-fitted by a nonlinear fit (Figure 2B-ii). This difference between the thiophenol plot and the functionalization molecules plot would appear to be the result of a relatively low Raman signal for the functionalized molecules in the mid-range (20–30 nm thickness) on top of the lack of large signals sub-20 nm, although large errors in Raman signal are noted in this thinner film range. These differences in the linearity of the fit can be explained by the variation in localized surface plasmon (LSPs) strength in discontinuous films (present in sub-20 nm gold surfaces) and the variation in surface roughness causing preferential adsorption for different molecules.^53^

An affinity test was run over the benzyl-terminated SAM and BNB-terminated SAM functionalized surface to demonstrate the influence of gold thickness on the detection of carbohydrates. We have assembled a proof-of-concept test for melezitose detection. Melezitose is a trisaccharide used as a preliminary affinity test with potential applications for other saccharides. This analyte is essential as an initial stepping stone for further specificity tests performed with other carbohydrates that can potentially trace early-stage cancer^13^ and, thus, as a new diagnostic platform. The functionalized SERS substrates were subsequently used to establish the specific fingerprint spectra and quantitatively determine carbohydrate affinity using PCA and SOM analysis of the different surfaces before and after immersion in the melezitose solution.

**Figure 3:**
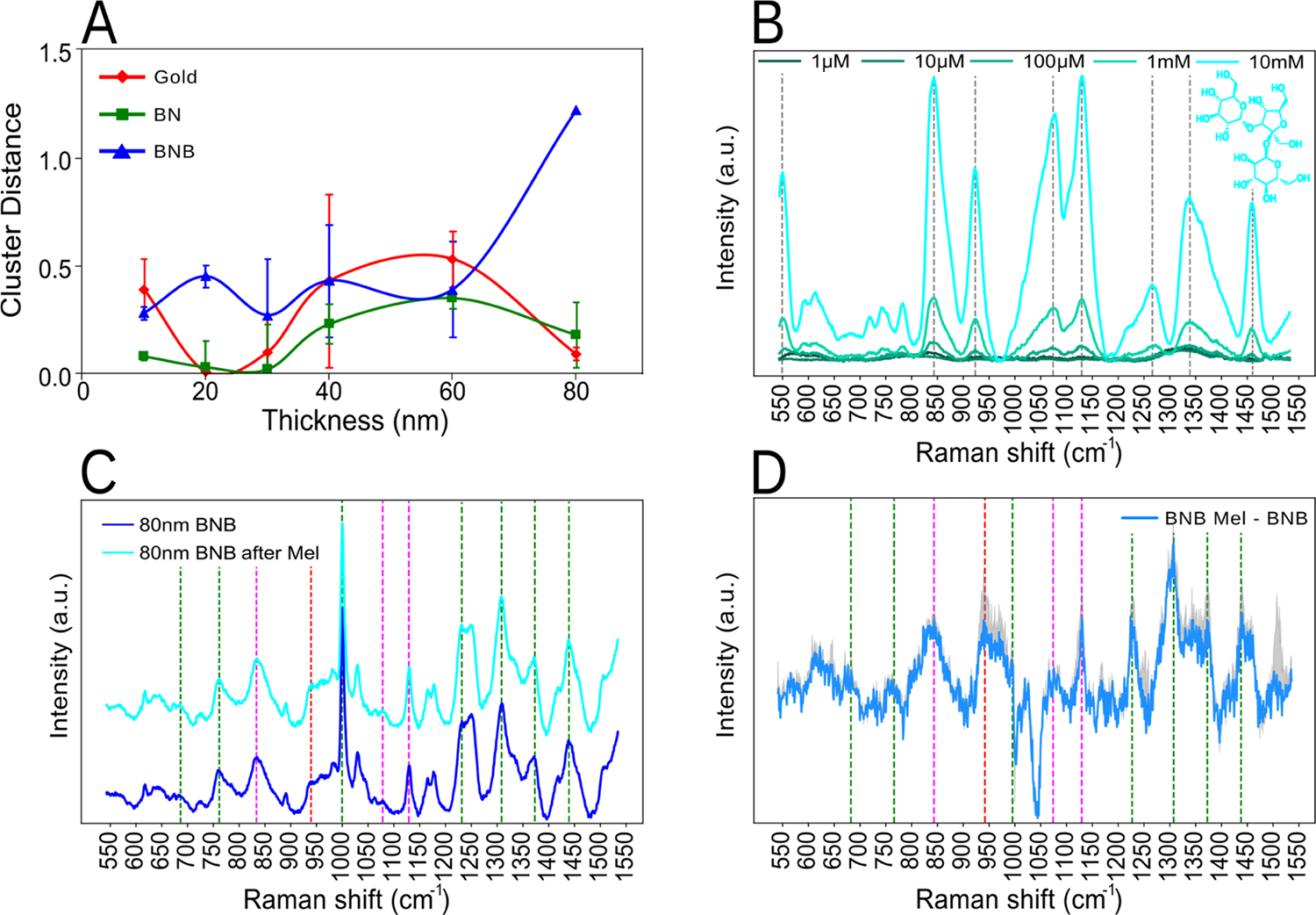
(A) Euclidian distance for the different PCA clusters between the prior to and post-melezitose submersion for gold, benzyl-terminated SAM (BN), and benzoboroxole-terminated SAM (BNB) for various thicknesses. The most significant separation is observed for the 80 nm thickness on BNB. (B) Raman spectra for different concentrations (0.001–10 mM) of melezitose (on aluminum) with the relevant peaks of melezitose highlighted by a gray dotted line. (C) Average Raman spectrum of BNB before and after melezitose submersion for the 80 nm gold thickness. (D) BNB Raman spectra difference between before and after melezitose at 80 nm gold, with the standard deviation outlined. Common peaks between the BNB and BNB after melezitose (green) melezitose, BNB and BNB after melezitose (magenta) and melezitose and BNB after melezitose (red).

**Figure 4:**
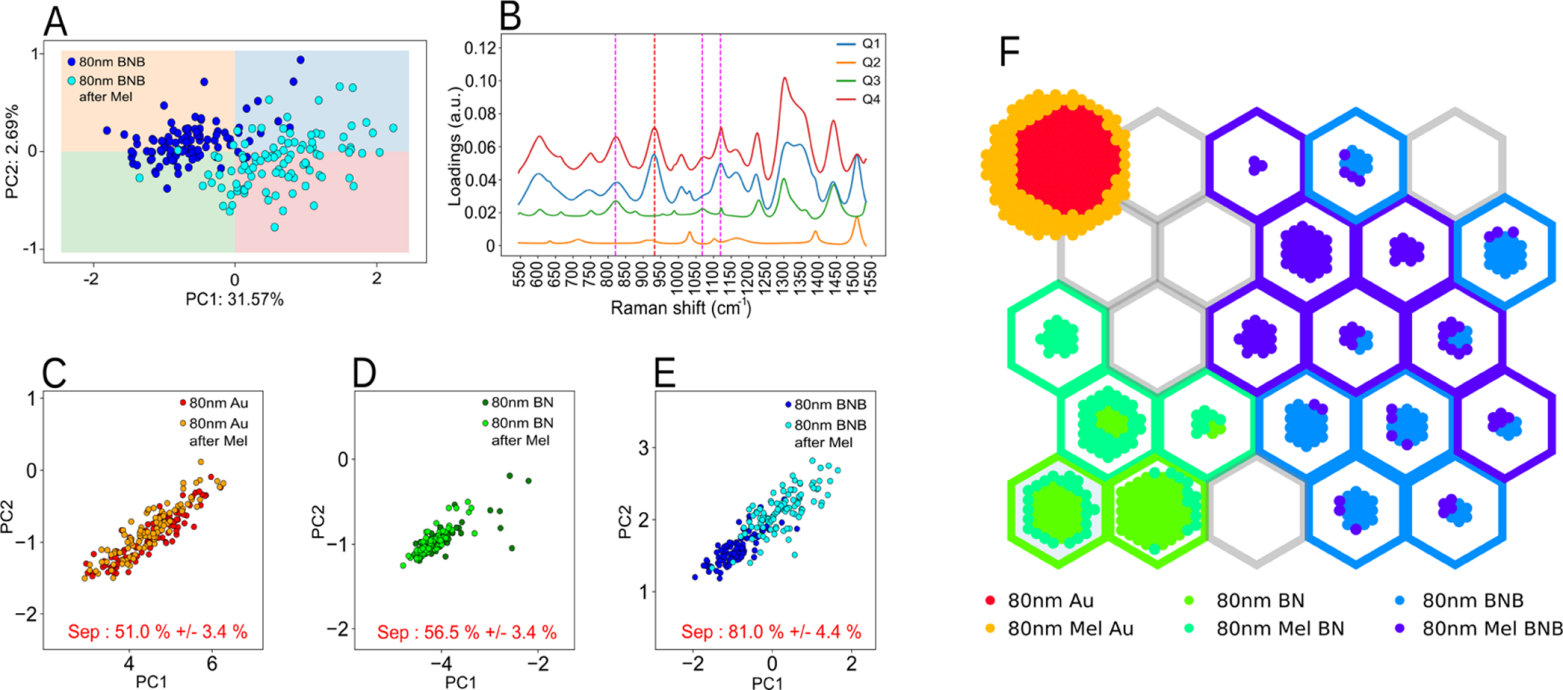
(A) PCA for 80 nm gold functionalized with benzoboroxole-terminated SAM (BNB) before and after melezitose (Mel) incubation. (B) PCA loading spectra for the different quadrants (Q1, Q2, Q3, and Q4) with the melezitose peak highlighted in dashed red and magenta. (C) PCA with *k*-NN cluster classification for the different functionalizations at 80 nm. Separation accuracy between clusters is considerably higher for the (E) benzoboroxole-terminated SAM functionalization (BNB), 81% score, relative to the 50% for (C) gold and 55% for (D) benzyl-terminated SAM (BN). (F) SOM clustering of spectra from the different substrates, including gold (Au), gold functionalized with benzyl-terminated SAM (BN), and gold functionalized with benzoboroxole-terminated SAM (BNB) for before and after melezitose (Mel) incubation.

It is evident from Figure 3A that the overall PCA cluster separation—the Euclidean distance between mean cluster positions—changes more significantly for the 80 nm gold thickness surfaces for BNB functionalization. Contrariwise, both benzyl-terminated SAM and gold cluster distances decrease to nearly 0 at 80 nm thickness in the PC space, indicating that the SERS spectra for both clusters are similar before and after immersion in melezitose. The PCA cluster distance between these surfaces at 80 nm is shown in Figure 4C–E. This difference is further demonstrated by performing a *k*-NN classification algorithm between the different classes (i.e., gold, BNB-terminated SAM, and benzyl-terminated SAM) before and after the melezitose submersion. While the cross-validation accuracy of the *k*-NN, identifying each melezitose submersion stage, shows an overall 50% accuracy in correctly determining the different states for gold and benzyl-terminated SAM, the algorithm shows 80% accuracy in the case of BNB-terminated SAM, distinguishing the different stages, thus establishing the functionalization potential for carbohydrate binding.

Moreover, we validated the gold thickness effect for sensing applications, specifically detecting melezitose. Melezitose was studied via Raman spectroscopy for a range of concentrations (0.001–10 mM), with the characteristic fingerprint spectra shown in Figure 3B. Further, an in-depth peak analysis was performed to detect melezitose in the post-carbohydrate immersed BNB-terminated SAM substrate (Figure 3C), identifying slight changes in the peaks with red and magenta dashed lines. The same peak variations can be accentuated by comparing the difference between the two spectra (Figure 3D). Additionally, the difference between the two spectra was extended to gold and gold after melezitose and benzyl-terminated SAM as well as benzyl-terminated SAM after melezitose, for all peaks listed in Table 3 comparing the two conditions (before and after melezitose incubation). This shows a predominantly high significance (*p* < 0.0001) for BNB-terminated SAM compared to gold and benzyl-terminated SAM, which showed no statistical significance for most peaks (Figure S7). The PCA results from Figure 3A for the BNB-terminated SAM before and after melezitose incubation at 80 nm gold thickness (Figure 4A) were further interrogated. The analysis was split into the four principal components (PC) quadrants (Q), Q1, Q2, Q3, and Q4. We extract the loadings from the PCA analysis to visualize quadrant differences (Figures S8 and S9), with dark and light gray representing positive and negative loading values, respectively. The resulting loadings from different quadrants are shown in Figure 4B. The different quadrant loading peaks were subsequently compared with the melezitose peaks, and the matching ones were highlighted with red and magenta dashed lines. Combining the identified peaks from Figures 3B–D and 4B, the resulting identified melezitose peaks are found to be at 843 ± 7, 940 ± 10, 1074 ± 5, and 1130 ± 7 cm^–1^. These are attributed to the stretching vibrations of the C–C and C–H, deformation vibrations of C–H and bending vibrations of C–O–H, bending vibrations of the C–H and C–O–H bonds, and bending and wobble vibrations of the CH and −OH groups,^54^ accordingly. The Raman peak analysis and the PCA results provide strong evidence that melezitose has bonded to the BNB-terminated SAM compared to the other surfaces that do not show the same separation.

**Table 3:**
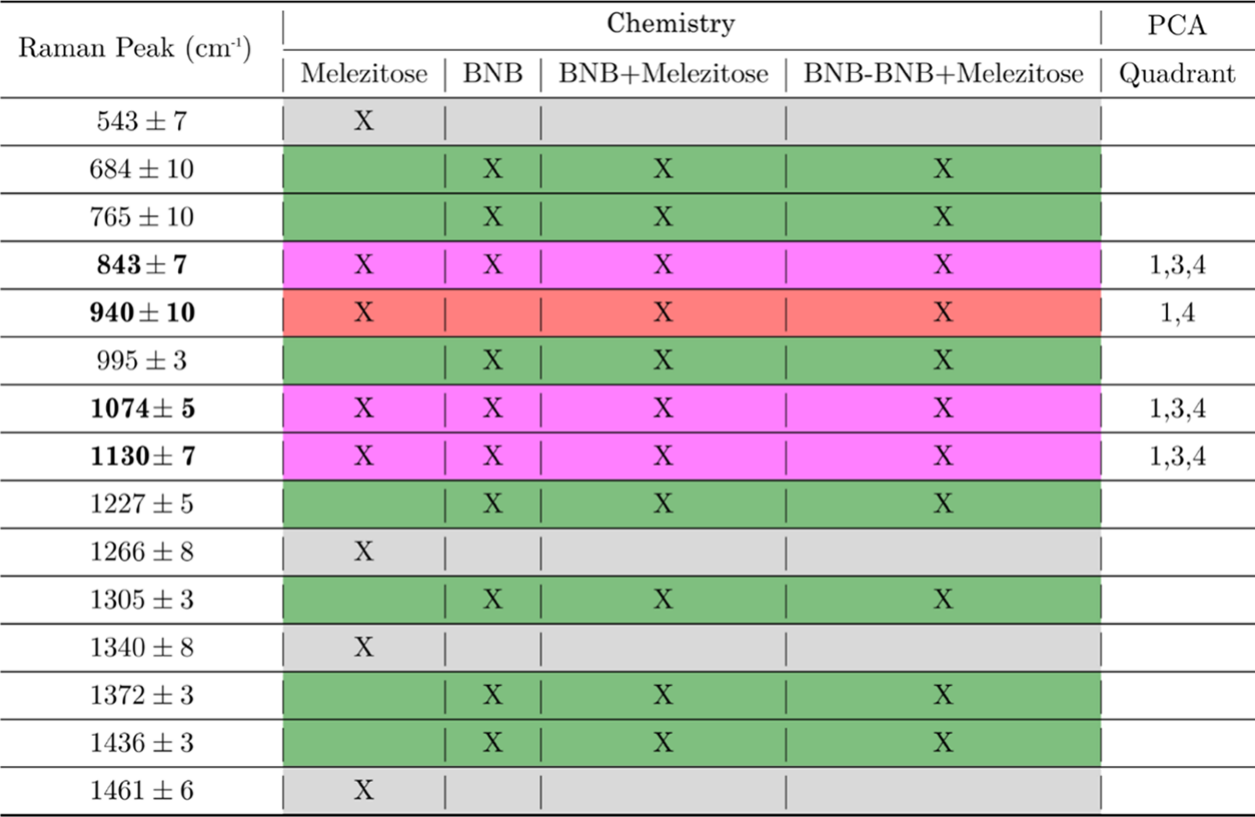
Raman Peaks Related to the Different Functionalizations over the Gold Substrate: Melezitose, Benzoboroxole-Terminated SAM (BNB), Benzoboroxole-Terminated SAM after Melezitose Incubation (BNB + Melezitose), Benzoboroxole-Terminated SAM Minus Benzoboroxole-Terminated SAM after Melezitose (BNB – BNB + Melezitose), and the PCA Loadings per Quadrant^a^

a The most prominent peaks have been assigned to 843 ± 7, 940 ± 10, 1074 ± 5, and 1130 ± 7 cm^–1^ as the main peaks’ differentiation between benzoboroxole-terminated SAM and benzoboroxole-terminated SAM after melezitose, with particular importance to 940 cm^–1^. The color scheme is related to the dashed lines in Figures 3C,D and 4B.

From the PCA in Figure 4A, there are prominent bands that the PCA is using to separate the datasets that are not normally associated with melezitose. Some of these bands could be due to local density inhomogeneity of the BNB-terminated SAM molecules, conformal change to the BNB-terminated SAM or melezitose, and induced orientation effects from BNB-terminated SAM-melezitose interaction, which needs further study. The appearance of other peaks not associated with melezitose is also verified by peak differences in Figure 3D around 684 cm^–1^, 765 cm^–1^, 995 cm^–1^, 1227 cm^–1^, 1305 cm^–1^, 1372 cm^–1^, and 1436 m^–1^; these are peaks that have more expression for BNB-terminated SAM after melezitose but are not related to melezitose peaks. There are also some peaks that are related to all three of the cases (melezitose, BNB-terminated SAM, and BNB-terminated SAM after melezitose) 843, 1074, and 1130 cm^–1^. Furthermore, one peak shows more prominence after the melezitose incubation over BNB-terminated SAM, related to melezitose at 940 cm^–1^. This prominence is also observed in the PCA loadings in Figure 4B since the 940 cm^–1^ peak is only shown for quadrants 1 and 4 of the loadings, which are mainly populated by BNB-terminated SAM after the melezitose samples. In contrast with the other three peaks, 843, 1074, and 1130 cm^–1^ are observed in quadrant 3, mostly populated by BNB-terminated SAM before the melezitose sample. It is also important to note the absence of quadrant 2 in any of these peaks, which is more populated with BNB-terminated SAM samples. All of the identified peaks are summarized in Table 3.

Additionally, a self-organizing map (SOM) was used to analyze this data. The SOM in Figure 4F shows a clear separation between the different surface functionalizations, including gold (red and yellow), benzyl-terminated SAM (green and lime), and benzoboroxole-terminated SAM (light blue and purple). The different colors represent the before and after melezitose submersion. Since both gold and benzyl-terminated SAM have no affinity to melezitose, their different states are closely clustered. On the other hand, BNB-terminated SAM has an affinity to melezitose, and thus, the clustering is more scattered, with the before and after melezitose submersion classified as two clustering sites, the light blue (before) and the purple (after). The BNB-terminated SAM sensitivity toward melezitose is in-line with the observations found via the PCA analysis (Figure 4A–E) and Raman spectral analysis (Figure 3B–D), confirming the need for thicker (80 nm) and rougher (7.2 nm) gold films for surface functionalization and analyte sensing.

**Figure 5:**
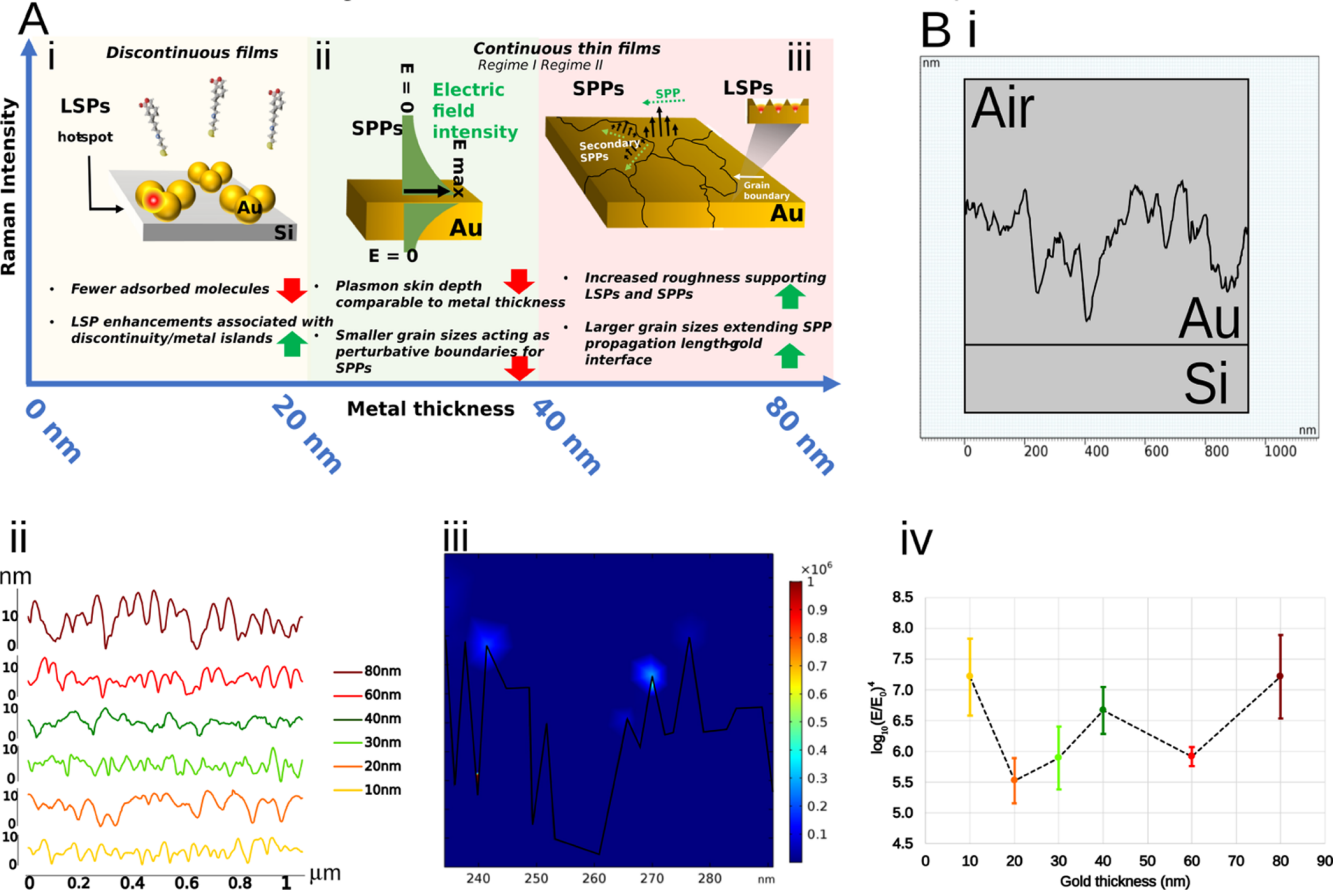
(A) Proposed dominant mechanisms responsible for the SERS intensity variations (i) in the low thickness regime (<20 nm), (ii) in the mid-range (20–40 nm), (iii) and thicker gold films (40–80 nm). Red and green arrows indicate effects that lower and increase the observed Raman signal. (B) Simulation of an AFM 2D profile. (i) Elements are sectioned into the silicon substrate (Si), the gold (Au), and the outside air layer. (ii) Example of the 2D AFM profiles for the different gold thicknesses. (iii) Enhancement field location in sharp edges and closed pockets. (iv) The common logarithm variation of the enhancing field for different gold thicknesses.

Various underpinning mechanisms may be responsible for the observed SERS enhancements for the 80 nm gold thickness, including surface functionalization and sensing trends, depicted in Figure 5A. Electromagnetic effects arising from plasmon-polaritons are the dominant mechanism;^55−57^ notably, these include LSP enhancement, most readily associated with SERS, where individual nanostructures support spatially localized plasmon-polaritons, or closely spaced surface features sustain high-energy LSPs in nanometric spaces, i.e., gap plasmons or SERS hot spots.^58−61^ Other possible effects are surface plasmon-polaritons at the grain boundaries as well as surface effects related to an increase in surface area for SAM adsorption due to the increased roughness and grain size for thicker films. Previous studies have shown that roughness creates defects in SAMs and influences their packing and organization, primarily resulting in more disordered SAMs.^62,63^ This effect can be compensated by an increase in grain sizes, as larger grain sizes create more ordered regions of the SAM.^64^ The 80 nm gold thickness surfaces are characterized by not only the highest roughness but also the largest grain sizes, thus opening the possibility for the latter to play a prominent role in promoting SAM quality and SERS enhancement. While roughened SERS substrates are a staple of SERS research,^65−67^ the prominence of such effects and their interplay, in the context of surface functionalization, would constitute interesting further study.

As an introductory measure, electromagnetic simulation was carried out to understand the plasmonic response of the surfaces with roughness geometries underpinned by (Figure 5B) AFM measurements. The different surfaces were thus imported into the COMSOL Multiphysics simulation platform and then analyzed using the wave optics module. The scattering field enhancement measurements were recorded and averaged over four different surfaces per thickness. A sample of each 2D AFM profile is shown in Figure 5B-ii. We can observe the difference in profile roughness with a small roughness oscillation for the 10 nm layer and larger oscillations when reaching the 80 nm thickness. The enhancing effect was observed at sharp points, i.e., the lightning rod effect, and at localized nanogaps (Figure 5B-iii). This form of enhancement is noticeable for different thicknesses, but it was more pronounced for the 80 nm gold. The different enhancing factors for different thicknesses are most notable at the 10 and 80 nm thick films (Figures 5B-iv, S10, and S11).

## Conclusions

We have systematically studied planar gold films of varying thicknesses and roughnesses and assessed the impact on molecular surface functionalization using the trisaccharide melezitose as an exemplar analyte.

An optimum gold layer thickness 80 ± 8 nm with 7.2 ± 1.0 nm *R*_RMS_ was established. This was evidenced via an algorithmic analysis with unsupervised machine learning methods in the form of PCA and SOM. The BNB-terminated SAM Raman spectra, pre- and post-melezitose application were analyzed in detail, and peaks were assigned. PCA demonstrated the clear advantage of 80 nm thick gold films functionalized with BNB-terminated molecules for melezitose detection with a significant increase in pre- and post-melezitose cluster separation in PCA scores space, which was further supported by a *k*-NN clustering analysis that returned a 30% increase in classification accuracy for the BNB-terminated SAM in contrast to the pre- and post-melezitose states for the pristine gold and benzyl-terminated SAM surfaces, respectively. Similarly, a SOM analysis displayed greater clustering separation for the two BNB-terminated states. Mechanisms to describe the intricacies of the signal variations observed have been introduced with prospection for further study. Electromagnetic models, based on experimentally acquired AFM topographies, were performed to offer a starting point for further theoretical analyses of plasmonic effects, with 10 nm and 80 nm thick films giving the largest enhancements.

While other affinity molecules tailored to detect various analytes will need to be considered separately, the general conclusions on the optimized conditions lay the platform to carefully design and fabricate advanced SERS active nanoarrays that will advance the development of portable lab-on-chip devices and sensors. These could be further functionalized with specific receptors for a range of analytes for multiplexed, highly specific, and sensitive detection while being selective for targeted biochemicals, detecting and analyzing a variety of target molecules pertinent for environmental, forensic, and biomedical applications.

## Supporting Information Available

Detailing the molecular surface functionalization steps, Raman peaks for the different functionalizations, AUC of the Raman spectra, intensity variation with gold film thicknesses, and statistical analyses (PDF)
