# Supplementary material for: Optimization of Nanosubstrates toward Molecularly Surface-Functionalized Raman Spectroscopy
Source: J Phys Chem C Nanomater Interfaces. 2022 Aug 8;126(32):13774–84. doi: 10.1021/acs.jpcc.2c03524 (PMC9393890; doi:10.1021/acs.jpcc.2c03524)
Supplement: Supplementary file 1 — jp2c03524_si_001.pdf [file jp2c03524_si_001.pdf]

-Supporting Information-

Optimization of Nanosubstrates Towards Molecularly Surface Functionalized Raman Spectroscopy

Paulo De Carvalho Gomes[1], Mike Hardy[1], Yazmin Tagger[1], Jonathan James Stanley Rickard[2], Paula Mendes[1] and Pola Goldberg Oppenheimer[1,3,]*

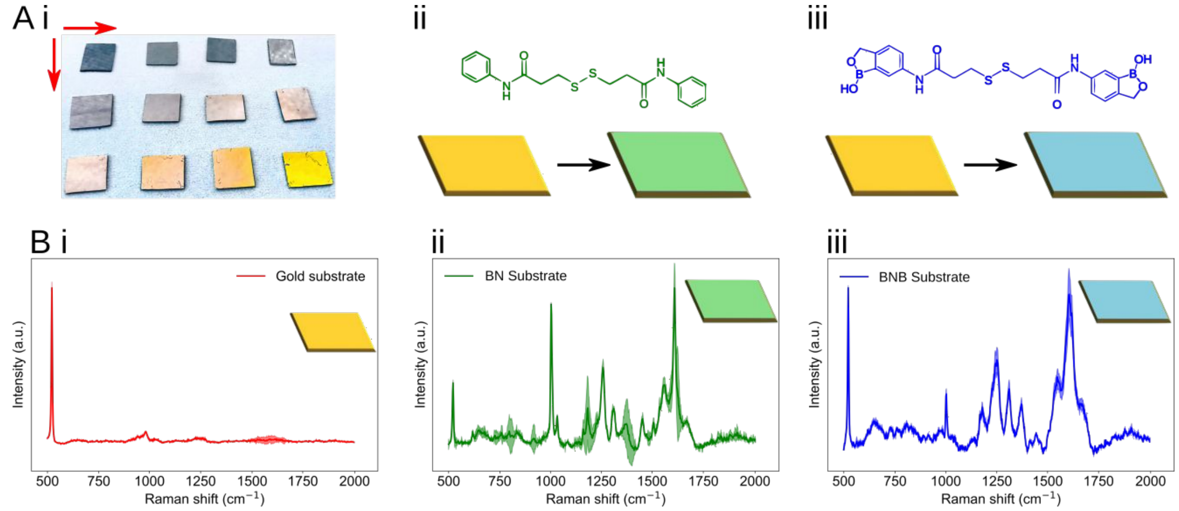

Figure S1. **(A)** Overview of the molecular surface functionalization steps on top of gold substrates **(i)** where the red arrow indicates the direction of increasing gold thickness. Gold functionalization including the **(ii)** benzyl-terminated SAM (BN) and **(iii)** benzoboroxole-terminated SAM (BNB). **(B)** Representative SERS spectra and standard deviation (shaded) of the 80 nm gold-coated substrates of **(i)** plain, **(ii)** BN and **(iii)** BNB.

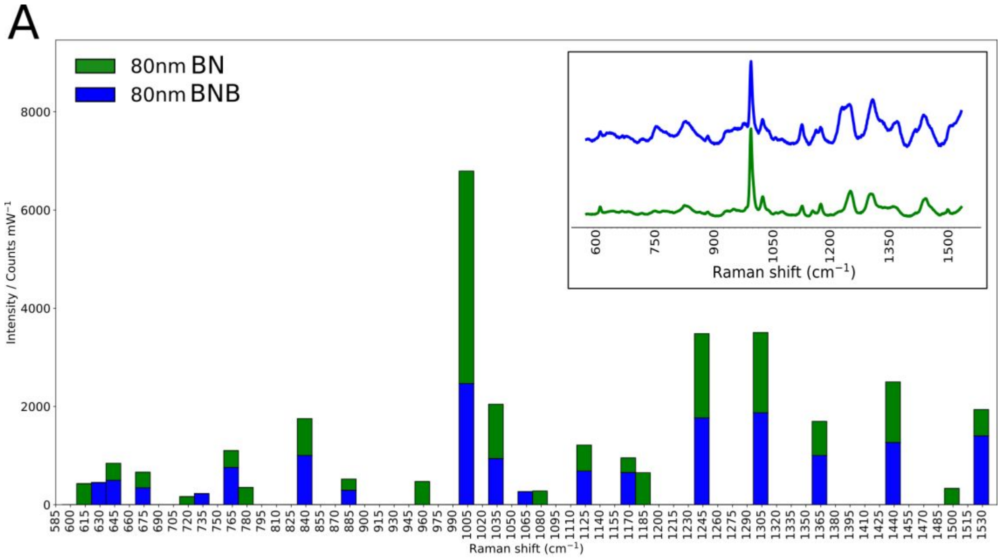

B

Raman shift (cm-1)	Functionalization	cont	Functionalization	cont.	Functionalization
619	BN	886	BNB	1176	BN
623	BNB	886	BN	1245	BNB
641	BNB	957	BN	1251	BN
647	BN	1000	BNB	1305	BN
675	BN	1000	BN	1308	BNB
679	BNB	1029	BN	1359	BN
723	BN	1030	BNB	1367	BNB
727	BNB	1060	BNB	1440	BNB
758	BN	1077	BN	1442	BN
763	BNB	1128	BN	1499	BN
781	BN	1129	BNB	1534	BN
835	BN	1164	BN	1536	BNB
836	BNB	1173	BNB		

Figure S2. Raman peaks for the different functionalizations including, the benzyl-terminated SAM (BN) (green) and benzoboroxole-terminated SAM (BNB) (blue). The peaks are mostly to carbon-carbon interactions, sp^2 and sp. Carbon-oxygen and carbon-nitrogen are also relevant characteristic peaks. **(A)** Peaks for the different surface functionalizations, with a bin size of 15cm^{-1}. **Inset.** Representative Raman spectra of BN and BNB at 80nm gold thickness. **(B)** Summary of

the Raman peaks in the bar plot in **(A)**. Peak assignments include: ν(C-O-C) 800-970cm^{-1}, ν(C-O-C) asym 1060-1150cm^{-1}, ν(C-C) alicyclic, aliphatic chain vibrations 600-1300cm^{-1}, ν(C-C) aromatic ring chain vibrations 1580cm^{-1}, 1600cm^{-1}, 1450cm^{-1}, 1500cm^{-1}, 1000cm^{-1}, δ(CH_3) 1380cm^{-1}, δ(CH_2) and δ(CH_3) asym 1400-1470cm^{-1}, ν(C=C) 1500-1900cm^{-1}.

Both *n* and *k* plots exhibit similar trends with an *n* minimum at 525 nm and a maximum at 680 nm wavelength and the extinction coefficient, with a minimum at 500 nm and a maximum at 620 nm wavelength. At the 633 nm laser excitation wavelength, the values were found to be n=1.10±0.06 and k=2.88±0.40 for planar gold, n=1.04±0.04 and k=3.09±0.39 for the benzyl-terminated SAM and n=1.18±0.20 and k=2.97±0.44 for the benzoboroxole-terminated SAM for the largest gold thickness setting. We have observed that *n* decreases with the increasing gold thickness until reaching 60 nm and then increases at the 80 nm thickness. There is an emergence of a trough at 530 nm wavelength that shifts slightly with the increase in gold thickness. A crest appears at 630 nm wavelength for sufficiently thick films, as shown from the increase at the 80 nm thickness in the bar plot (**Fig. S3D**). Moreover, the *k* value increases until reaching 30 nm and decreases until 80 nm (**Fig. S3E**). The calculated gold roughness from the ellipsometry measurements is 8.9±3.1 nm, which is in good agreement with the value obtained from the AFM measurements (**Fig. 1B**) for the 80 nm gold layer.

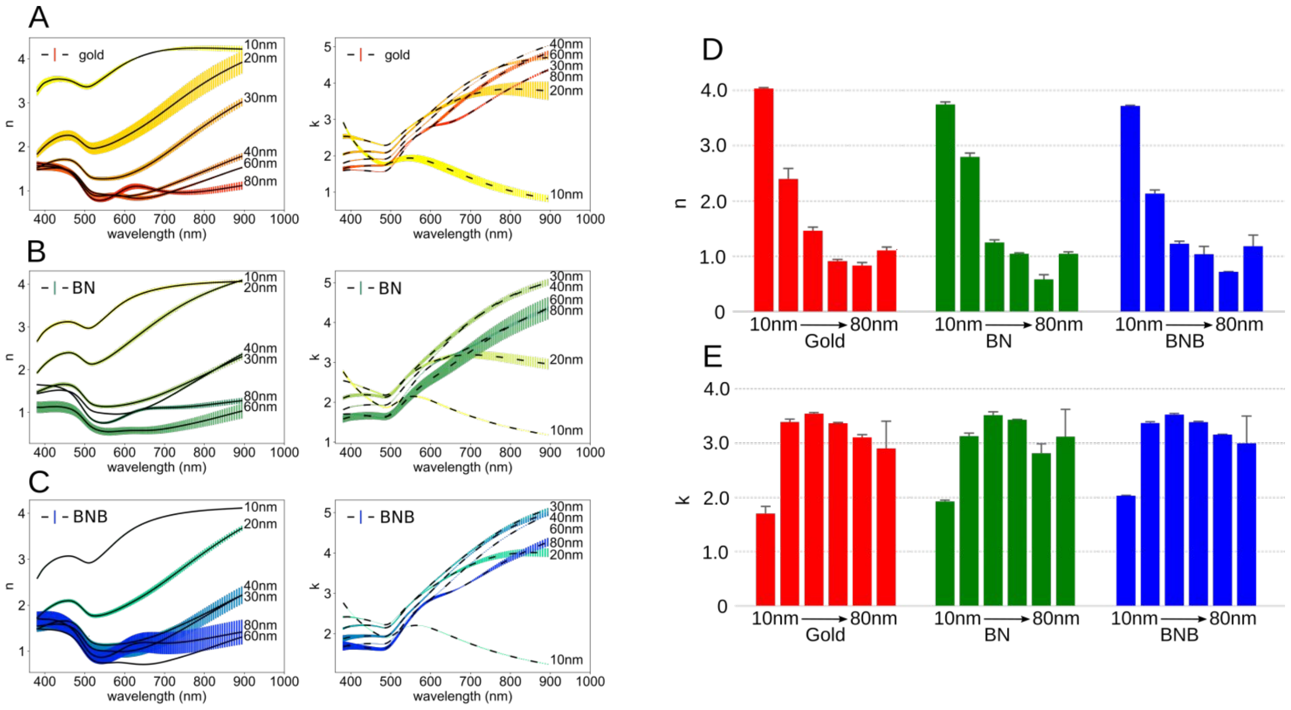

Figure S3. Refractive index and extinction coefficient of planar gold **(A)** and functionalised substates with **(B)** BN and **(C)** BNB with the corresponding **(D)** *n* and **(E)** *k* values at the wavelength of 633 nm.

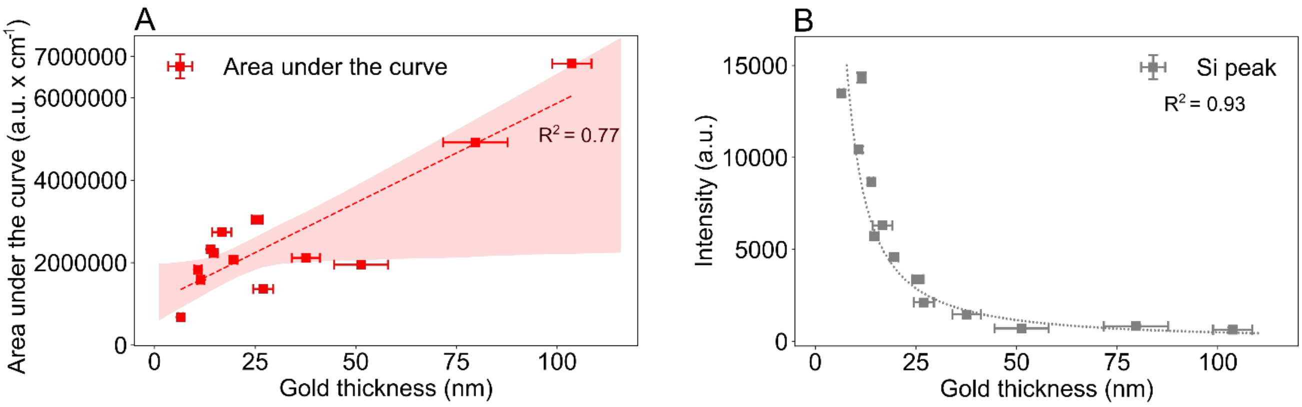

Figure S4. (A) AUC of the Raman spectra increases with increasing gold thickness. **(B)** Raman intensity of the silicon peak at ~520.5cm^{-1} decreases with the increasing gold thickness.

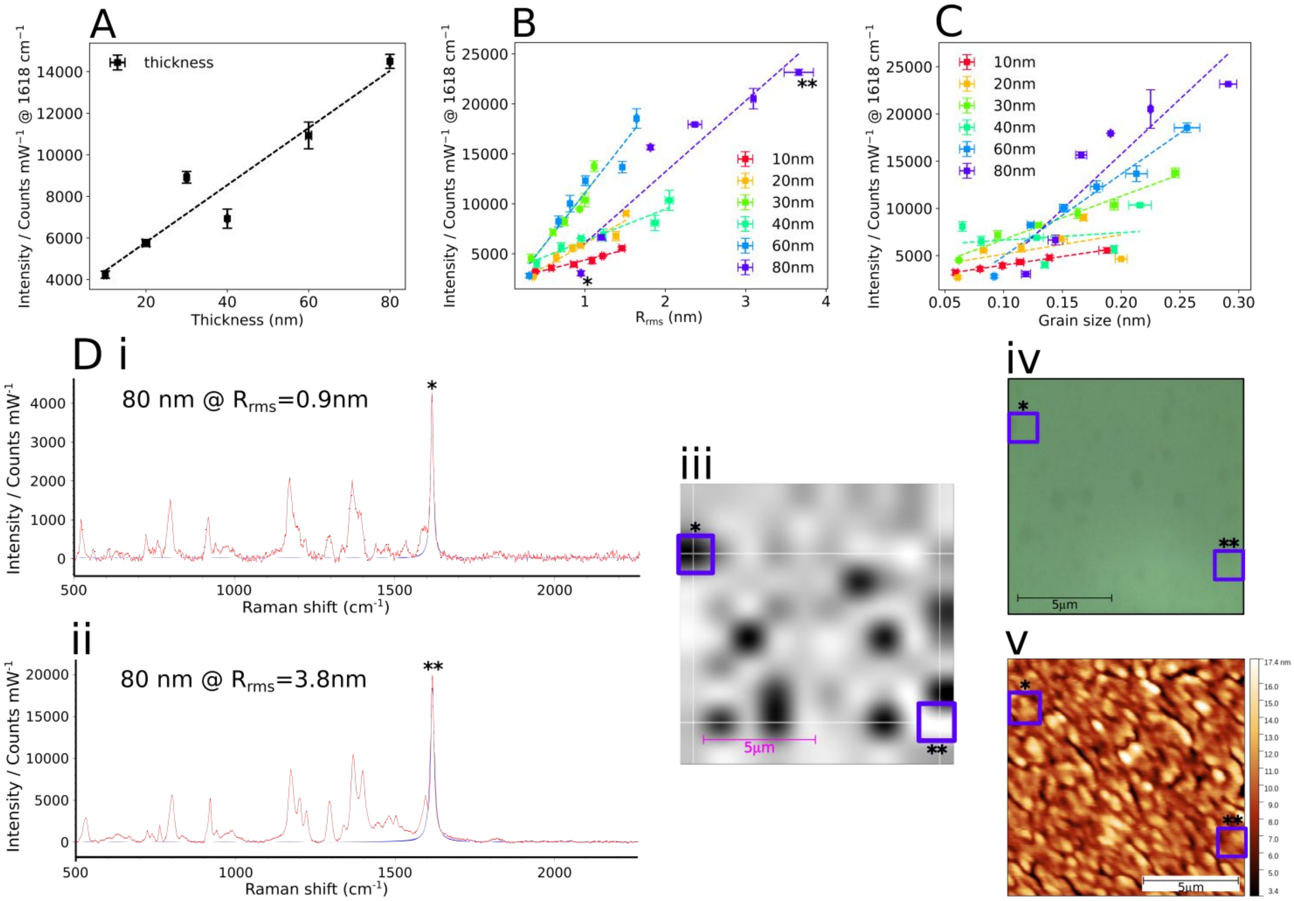

Figure S5. (A) Intensity variation with gold film thicknesses. Showing an intensity increase for the thiophenol peak for an increase in thickness. **(B)** Intensity variation with gold film RMS roughness for different thicknesses. Showing a consistent increase of the thiophenol intensity peak for both increases in roughness and thickness **(D)** Intensity variation between two different roughness values from representative Raman maps with **(i)** RMS value of 0.9nm an intensity value of 4,000 counts, compared to the **(ii)** 20k counts for RMS value of 3.8nm. The representative Raman map shown in **(iii)** highlights the two different spots and the respective peak intensity with the corresponding RMS values. In each case, an identical spot was observed and used under optical microscope **(iv)** and *via* the AFM **(v)**.

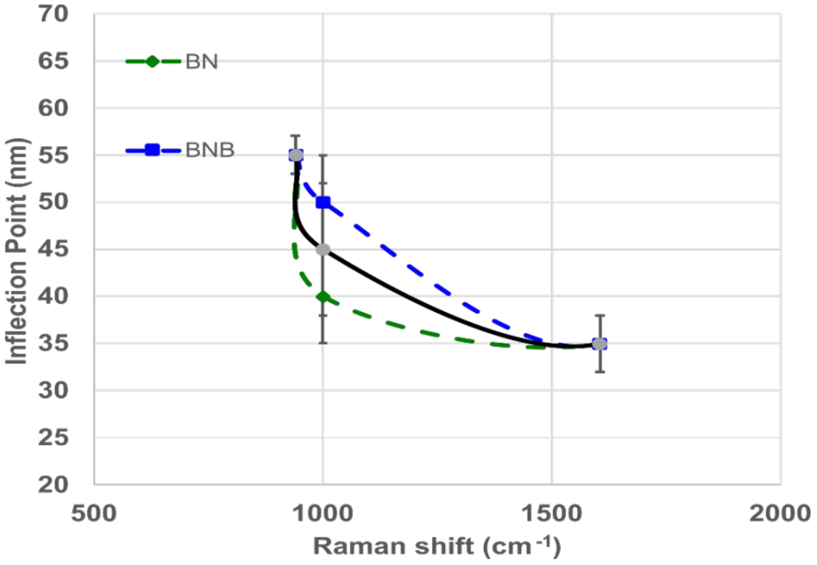

Figure S6. Inflection point representation for benzyl-terminated SAM (blue), benzoboroxole-terminated SAM (green) and the average trend (black).

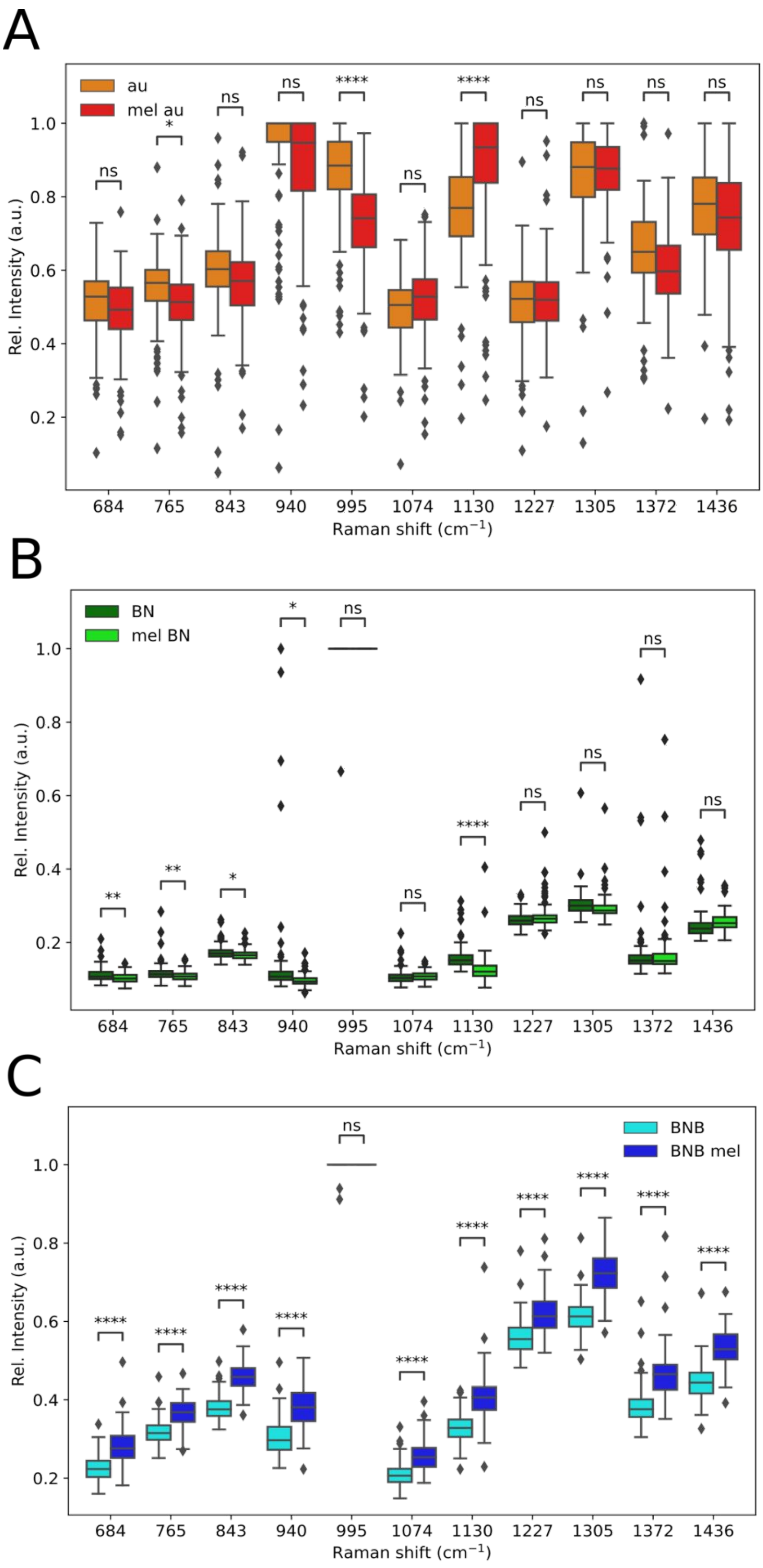

Figure S7. Box plots of Raman peaks for which multiple pairwise comparisons (Tukey's HSD) were performed in pairs examining the difference between **(A)** gold and with the melezitose, **(B)** BN and BN after melezitose and **(C)** BNB and BNB after melezitose substrates. Each spectrum was baseline subtracted using the asymmetric least square method and normalized between 0 to 1. The peaks were selected from **Table 3** (*i.e.,* 684, 765, 843, 940, 995, 1074, 1130, 1227, 1305, 1372, 1436 cm^{-1}) with the maximum intensity value extracted from these for statistical analysis. Statistically significant difference was observed for BNB and BNB mel for all peaks (p<0.001) except for the 995cm^{-1} peak, which coincided with the maximum in both normalized samples.

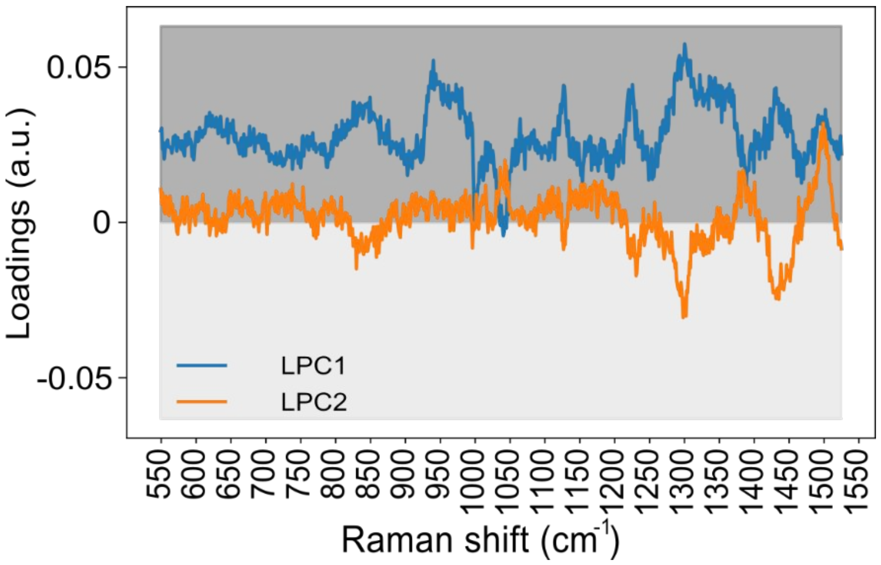

Figure S8. PCA loading plots for PC1 and PC2. Light grey and dark grey colours separate the positive and negative loading values to build up the quadrant Raman like spectra.

Three possible underpinning mechanisms may be responsible for the observed SERS enhancements, surface functionalization, and sensing trends. The mechanisms are film thickness, surface roughness, grain size and adsorbate complexity, which are connected to areal increase, molecular adsorption at the surface and the lightning rod effect (**Fig. 5A**).

The gold film thickness influences the dissipation of surface plasmon-polariton modes, which protrude a finite distance into the media from the surface interface. While this distance is lesser for the side of the medium with the higher refractive index, gold, there may be a perturbative effect from the substrate interface should the film thickness be comparable to the penetration depth of the laser. This value is often characterized as a "skin depth" whose intensity equals 1/e of the excitation's maximum value. Across the visible range in gold, the plasmon-polariton skin depth is 20-30nm [1]. This depth value is in qualitative agreement with minima in the plots in **Fig. 2B**, as indicated by the presentation of inflection points, which appear in the spectral mid-range. This minimum also appears on the simulation runs in **Fig. 5Biv**, where the enhancing factor had minima located between 20-30nm. However, we assign other effects to the gold film thickness in these substrates, focusing on the concurrent phenomena (**Fig. 5A**) rather than the specific effect of having thicker layers of gold.

Additionally, the rough surface features may support hot spots *via* the crowding of electric fields around sharp facets associated with the said features, *i.e.,* the lightning rod effect [2] was demonstrated from the COMSOL simulations (**Fig. 5Biii**). However, the effects are damped on average, so it should not affect the overall average SERS signal much.

There is a further relationship between surface nano-roughness and film thickness. The roughness increases monotonically by approximately a factor of eight from 5-80nm thick gold films (**Fig. 1B**). Here, we quantify the roughness by the well-known arithmetic mean square roughness, R_q:

$$R_q = \sqrt{\frac{1}{N}\sum_{n=1}^{N}(z_n - \dot{z})^2} \qquad [2]$$

Where z_n is the horizontal distance along the *x*-axis over which surface profile measurements occur, intuitively, rougher films constitute an increase in the surface area. Analogously, S_q is useful to represent a 2D area roughness (as opposed to 1D line scan measurements), characterized as an areal increase factor (= actual area/apparent area). Calculating the areal increase by using a triangulation method (**Fig. S8**).

$$A_{12} = \frac{h_x h_y}{4} 1 + \sqrt{\left(\frac{z_1 - z_2}{h_x}\right)^2 + \left(\frac{z_1 + z_2 - z_{av}}{h_y}\right)^2} \qquad [3]$$

Where h_x and h_y correspond to pixel dimensions, this suggests that an increase in surface area may only partially mediate the enhancement mechanism.

Overall, the areal increase results in a 50% increase with a measured R_q increase by a factor of 8 [Eq. 2] [Eq. 3] (hx/y = 3, z_1 = 1→2; z_2=1).

Furthermore, grain size can also explain the behavior observed between thiophenol, benzyl-terminated SAM and benzoboroxole-terminated SAM. Sputtered gold grain sizes increase with thickness, and according to the 2D van der Drift model introduced by Dammers, the relationship between film thickness (*d*) and grain size (*D*) is:

$$D \propto \sqrt{d} \qquad [4]$$

Metal grain sizes tend to increase with film thickness [2,3] and have several possible impacts on the Raman signal. First, the grain boundaries may have abnormal adsorption properties at the surface, affecting the number of analyte molecules adsorbed at the surface and subsequent Raman signal [4]. We note that the increases in the AUC with the increasing gold thickness (**Fig. 1A, C** and **Fig. S4A**) are attributed to increased fluorescence from increased numbers of gold molecules. The optical properties of the functionalized gold films are comparable (barring *n* and *k* for the thinnest films), indicating little change in molecular adsorption as film thickness varies (**Fig. S3 D** and **E**).

Moreover, comparing the different functionalization molecule sizes and complexities, thiophenol (**Fig. 1D** inset) is relatively smaller than benzyl-terminated SAM (**Fig. S1Aii**) and benzoboroxole-terminated SAM (**Fig. S1Aiii**). This fact influences how adsorption happens on different gold thicknesses and, subsequentially, roughness and grain size. In **Fig. 1B** and the SERS intensity in **Fig. 1D**, both roughness measurements show a linear trend for different gold thicknesses for thiophenol. However, for the benzyl-terminated SAM and benzoboroxole-terminated SAM, the roughness measurements are linear, but SERS intensity in **Fig. 2B** shows an inflection point near the 30 nm thickness, and only after this threshold thickness does the SERS signal increase. From these observed facts, we can deduce that large molecules require different surface conditions for good adsorption than simpler molecules like thiophenol. Thus, for planar gold thickness above the 30 nm thickness the gold surface has the proper roughness and grain size conditions to support benzyl-terminated SAM and benzoboroxole-terminated SAM growth, which justifies the SERS signal's appearance after this thickness. However, we have also shown that it is not sufficient to have a noticeable SERS signal from the functionalization to be able to use the surfaces as sensing devices, as observed by the melezitose test performed in **Fig. 3A** and support analysis of PCA. The clustering difference, which checks how similar two different SERS spectra are, shows that it was not until 80 nm thickness that there was some cluster separation, i.e., proper surface functionalization by having the molecules well adsorbed and oriented. 80 nm thickness of gold possesses the required roughness and grain size to support functional surface functionalization. It has acquired sensing capabilities, which are later observed in more detail from the SOM analysis (**Fig. 4F**), highlighting the functionalization and function of the benzoboroxole-terminated SAM. The defining factor in the SERS signal among these different surface functionalizations is the adsorbate size (**Fig. S1Aii** and **Fig. S1Aiii** vs **Fig. 1D**). A small, adsorbed permits easier contact and growth for any thickness and roughness, and a more complex large adsorbate requires the proper surface condition for both growth and function.

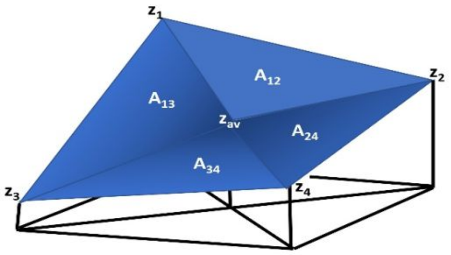

$$A_{12} = \frac{h_x h_y}{4}\sqrt{1+\left(\frac{z_1-z_2}{h_x}\right)^2+\left(\frac{z_1+z_2-2\bar{z}}{h_y}\right)^2}$$

$$A = A_{12} + A_{23} + A_{34} + A_{41}$$

Figure S9. Calculation of actual surface area by triangulation. **(Left)** *Z*-points are height measurement locations and A-planes are projected regions of areal increase. The surface area is estimated as following: z_i for i = 1, 2, 3, 4 denotes values in four neighbour points (pixel centres), and h_x and h_y pixel dimensions along corresponding axes. If an additional point is placed in the centre of the rectangle which corresponds to the common corner of the four pixels (using the mean value of the pixels), four triangles are formed, and the surface area can be approximated by summing their areas. Therefore, for the area of one triangle (top) and the surface area of one pixel (bottom) yields the formulas used for the calculation **(right)**.

Multiple pairwise comparisons (Tukey's HSD) relating the different conditions in pairs was performed. Each sample included 100 Raman signal points. The intensity value was taken from the maximum value between the 1575 and 1625cm^{-1} range to accommodate the 1618cm^{-1} peak for thiophenol (thio) and the 1605cm^{-1} for the functionalisations (BN and BNB). For the thickness variations (**Figure S10**), it was found that there is a statistically significant difference for thiophenol for all thicknesses (p<0.001) (**Fig.S10, D**). Similar results were obtained for the benzoboroxole-terminated SAM (**Fig. S10, C**) however, a lower significance was obtained with 10nm and 20nm gold thicknesses (p<0.5) and no significant difference was observed between the 30-60nm and 40-60nm thicknesses (p<0.9 and 0.002, respectively). For the benzyl-terminated SAM (**Fig. S10, B**), only the 80nm thickness showed significant difference (p<0.001) and thinner gold showed less significance with the lowest found to be between 30-60nm, 40-60nm and 30-40nm (p=0.9). Interestingly, both benzyl-terminated SAM and benzoboroxole-terminated SAM exhibit a lower significance in this region and there is a very small difference in the

Raman signal around this thickness, reaffirming the presence of an IP. For gold (**Fig. S10, A**), there is a low statistical significance for different thicknesses (p=0.1).

A

thickness1	thickness2	p-value	sample
10	30	0,001	au
10	80	0,002	au
30	60	0,048	au
30	40	0,089	au
60	80	0,184	au
10	20	0,212	au
20	30	0,254	au
40	80	0,289	au
10	40	0,477	au
20	80	0,571	au
10	60	0,610	au
20	40	0,900	au
20	60	0,900	au
30	80	0,900	au
40	60	0,900	au

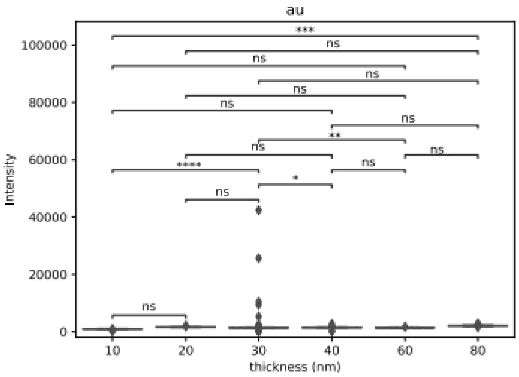

B

10	80	0,001	BN
20	80	0,001	BN
30	80	0,001	BN
40	80	0,001	BN
60	80	0,001	BN
10	30	0,021	BN
10	40	0,109	BN
10	60	0,213	BN
10	20	0,236	BN
20	30	0,900	BN
20	40	0,900	BN
20	60	0,900	BN
30	40	0,900	BN
30	60	0,900	BN
40	60	0,900	BN

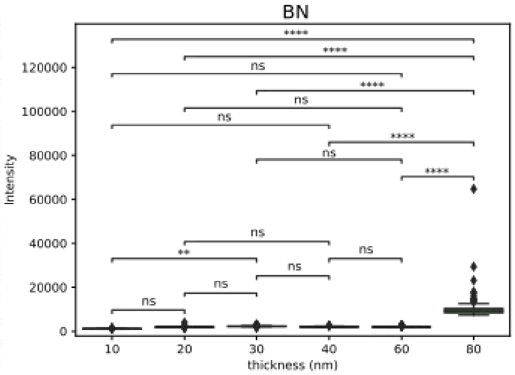

C

10	30	0,001	BNB
10	40	0,001	BNB
10	60	0,001	BNB
10	80	0,001	BNB
20	30	0,001	BNB
20	40	0,001	BNB
20	60	0,001	BNB
20	80	0,001	BNB
30	40	0,001	BNB
30	80	0,001	BNB
40	80	0,001	BNB
60	80	0,001	BNB
40	60	0,002	BNB
10	20	0,469	BNB
30	60	0,900	BNB

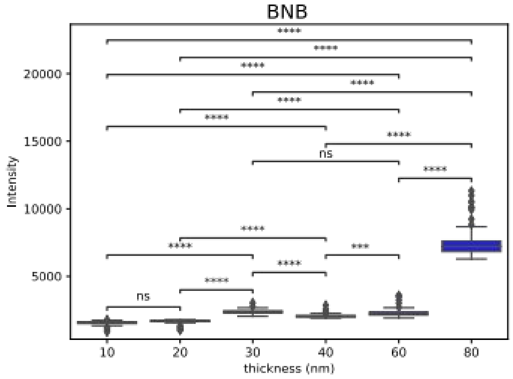

D

10	20	0,001	thio
10	30	0,001	thio
10	40	0,001	thio
10	60	0,001	thio
10	80	0,001	thio
20	30	0,001	thio
20	40	0,001	thio
20	60	0,001	thio
20	80	0,001	thio
30	40	0,001	thio
30	60	0,001	thio
30	80	0,001	thio
40	60	0,001	thio
40	80	0,001	thio
60	80	0,001	thio

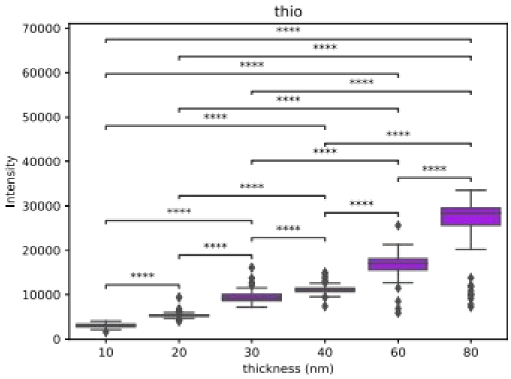

Figure S10. Statistical analysis of the Raman signal significance variation with thickness for **A)** gold, **B)** benzyl-terminated SAM, **C)** benzoboroxole-terminated SAM and **D)** thiophenol.

For the variation in functionalisation (**Figure S11**), we found a high statistically significant difference between all the different functionalisations and gold for 10nm (**Fig. S11. A**) and 80nm (**Fig. S11, F**) gold thickness (p<0.001). Similar was found for the other thicknesses (**Fig. S11, B**), although a low significance was identified for gold and benzoboroxole-terminated SAM for 60nm (**Fig. S11, D**) and between benzyl-terminated SAM and benzoboroxole-terminated SAM. Notably, the low significance found at a thickness of 30nm (**Fig. S11, C**) comparing gold with BN, gold with BNB and BN with BNB is related to the inflection point. This indicates that for the 30nm gold, there is no significant difference among gold, benzyl-terminated SAM and benzoboroxole-terminated SAM since due to the IP (*i.e.,* intensity minimum, where the Raman signal is low). For the 60nm (**Fig. S11, E**), there is low significance between benzyl-terminated SAM and gold and between benzyl-terminated SAM and benzoboroxole-terminated SAM (p=0.02 and 0.26, respectively).

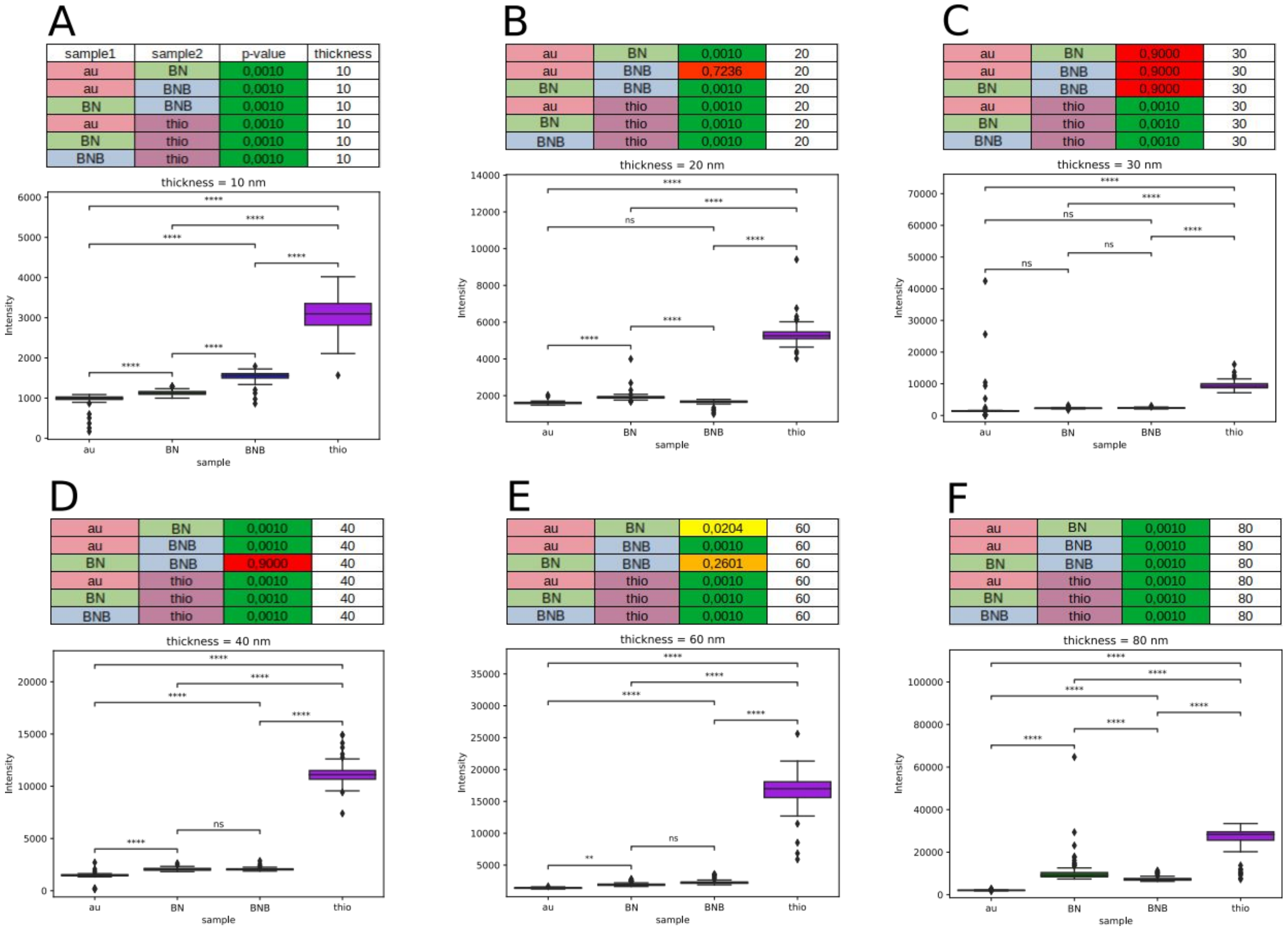

A

sample1	sample2	p-value	thickness
au	BN	0,0010	10
au	BNB	0,0010	10
BN	BNB	0,0010	10
au	thio	0,0010	10
BN	thio	0,0010	10
BNB	thio	0,0010	10

B

au	BN	0,0010	20
au	BNB	0,7236	20
BN	BNB	0,0010	20
au	thio	0,0010	20
BN	thio	0,0010	20
BNB	thio	0,0010	20

C

au	BN	0,9000	30
au	BNB	0,9000	30
BN	BNB	0,9000	30
au	thio	0,0010	30
BN	thio	0,0010	30
BNB	thio	0,0010	30

D

au	BN	0,0010	40
au	BNB	0,0010	40
BN	BNB	0,9000	40
au	thio	0,0010	40
BN	thio	0,0010	40
BNB	thio	0,0010	40

E

au	BN	0,0204	60
au	BNB	0,0010	60
BN	BNB	0,2601	60
au	thio	0,0010	60
BN	thio	0,0010	60
BNB	thio	0,0010	60

F

au	BN	0,0010	80
au	BNB	0,0010	80
BN	BNB	0,0010	80
au	thio	0,0010	80
BN	thio	0,0010	80
BNB	thio	0,0010	80

Figure S11. Statistical analysis of the Raman signal significance variation with functionalisation for gold thicknesses of **A)** 10nm, **B)** 20nm, **C)** 30nm, **D)** 40nm, **E)** 60nm and **F)** 80nm.

References

1 Homola, J. Surface Plasmon Resonance Based Sensors (Vol. 4). Springer-Verlag Berlin Heidelberg, 2006.

2 Dammers, A.; Radelaar, S. Two-dimensional computer modelling of polycrystalline film growth. *Text. Microstruct.* **1991**, *14*, 757-762.

3 Thompson, C. V. Grain growth in thin films. *Annu. Rev. Mater. Sci.* **1990**, *20*, 245-268.

4 Schultz, Z. D. Demystifying SERS: a newcomer's guide to using surface enhanced Raman scattering. *Spectroscopy*, **2020**, *35*, 15-19.
